# Approach towards total knee arthroplasty in Brazil: cross-sectional study

**DOI:** 10.1590/S1516-31802009000400003

**Published:** 2009-12-07

**Authors:** Raul Frankllim de Carvalho Almeida, Antônio Altenor Bessa de Queiroz, João Carlos Belloti, José Maria Bedran de Castro, Moisés Cohen, Ricardo Dizioli Navarro

**Affiliations:** 1 MD. Orthopedist and postgraduate student in the Department of Orthopedics and Traumatology, Universidade Federal de São Paulo (Unifesp), São Paulo, Brazil.; 2 MD, MSc. Orthopedist and Head of the Knee Group, Department of Orthopedics and Traumatology, Universidade Federal de São Paulo (Unifesp), São Paulo, Brazil.; 3 MD, PhD. Orthopedist and professor in Department of Orthopedics and Traumatology, Universidade Federal de São Paulo (Unifesp), São Paulo, Brazil.; 4 MD. Orthopedist and adjunct professor in the Department of Orthopedics and Traumatology, Universidade Federal de São Paulo (Unifesp), São Paulo, Brazil.; 5 PhD. Orthopedist and full professor in the Department of Orthopedics and Traumatology, Universidade Federal de São Paulo (Unifesp), São Paulo, Brazil.

**Keywords:** Knee, Knee joint, Arthroplasty, Knee prosthesis, Brazil, Joelho, Articulação do joelho, Artroplastia, Prótese do joelho, Brasil

## Abstract

**CONTEXT AND OBJECTIVE:**

Total knee arthroplasty (TKA) has evolved particularly since the 1970s, with improvements in implants and surgical instruments, and has thus become an effective intervention for treating knee arthrosis. Many studies have presented rates of satisfactory clinical and radiological results greater than 90%, from follow-ups of over ten years. Nevertheless, despite scientific evidence showing the efficacy of TKA, the approaches taken present controversies in certain respects. The objective of this study was to evaluate how the Brazilian orthopedists deal with TKA, with investigation of the main aspects of this procedure.

**DESIGN AND SETTING:**

Cross-sectional survey conducted during the 39^th^ Brazilian Congress of Orthopedics and Traumatology, in São Paulo, Brazil, in November 2007.

**METHODS:**

We applied a questionnaire to orthopedists registered at the congress. The questionnaire was randomly distributed and participation was voluntary; 858 completed questionnaires were included in the analysis.

**RESULTS:**

Most of the Brazilian orthopedists were members of SBOT and worked in the southeastern region. They used imported cemented implants through an anterior access route centered on the patella, with replacement of the joint surface of the patella and preservation of the posterior cruciate ligament. They did not have experience with simultaneous bilateral TKA. Postoperatively, they used antibiotics and suction drains for 48 hours. There was no consensus regarding prophylaxis for venous thromboembolism or the frequency of the main complications.

**CONCLUSION:**

The majority of Brazilian orthopedists work in the southeastern region of the country and agree about the main aspects of the approaches towards TKA.

## RESUMO

**CONTEXTO E OBJETIVO:**

A artroplastia total do joelho (ATJ) evoluiu sobremaneira desde os anos 70, com melhora dos implantes e do instrumental cirúrgico, tornando-se uma intervenção efetiva para o tratamento da artrose do joelho. Muitos estudos apresentam resultados clínicos e radiológicos satisfatórios superiores a 90% no acompanhamento acima de 10 anos. Apesar das evidências científicas sobre sua eficácia da ATJ, a sua abordagem apresenta controvérsias em alguns aspectos. O objetivo do estudo foi avaliar como o ortopedista brasileiro aborda a ATJ e os principais aspectos técnicos na realização deste procedimento.

**TIPO DE ESTUDO E LOCAL:**

Estudo transversal, realizado durante o 39^o^ Congresso Brasileiro de Ortopedia e Traumatologia em São Paulo, Brasil, em novembro de 2007.

**MÉTODOS:**

Aplicamos um questionário aos ortopedistas inscritos no congresso. A distribuição foi aleatória com adesão voluntária. Foram incluídos 858 questionários para análise.

**RESULTADOS:**

A maioria dos Ortopedistas Brasileiros são membros da SBOT e atua na região sudeste. Usam o implante importado, cimentado, por via de acesso anterior centrada na patela, com substituição da superfície articular da patela e preservação do ligamento cruzado posterior e não tem experiência com a artroplastia total bilateral simultânea. No pós-operatório utilizam antibióticos e dreno de sucção por 48 horas. Não houve consenso quanto à profilaxia para tromboembolismo venoso e frequência das principais complicações.

**CONCLUSÃO:**

A maioria dos ortopedistas brasileiros trabalha na região sudeste e concorda quanto aos principais aspectos da abordagem da ATJ.

## INTRODUCTION

Total knee arthroplasty (TKA) has evolved particularly since the start of the 1970s, with improvements in the quality of implants and surgical instruments, and has thus become an effective intervention for treating knee arthrosis if conservative treatment fails. Many studies have presented rates of satisfactory clinical and radiological results greater than 90%, from follow-ups of over ten years.^1^^,^^2^^,^^3^^,^^4^^,^^5^

Although it is an effective surgical procedure,^1^^,^^2^^,^^3^^,^^4^^,^^5^^,^^6^^,^^7^^,^^8^^,^^9^^,^^10^ several aspects of the approaches taken towards TKA present controversy. Among these are the choices of access route, use or nonuse of orthopedic bone cement, preservation of the posterior cruciate ligament, replacement of the joint surface of the patella, whether to carry out bilateral procedures in a single operation or in two operations at different times, and the duration of prophylaxis against infections and venous thromboembolism, among others.

According to the Brazilian Institute for Geography and Statistics (IBGE),^11^ Brazil will have the sixth largest population of elderly people in the world in 2030, thereby making TKA a procedure increasingly used in Brazilian orthopedic practice. Because of the success of TKA and the controversies that exist regarding various aspects of the approaches taken, we proposed to carry out this cross-sectional study on the use of TKA in Brazil. The main aspects of how this surgical procedure is performed and its main complications were analyzed. The parameters guiding us were previous cross-sectional studies published in Brazil.^12^

## OBJECTIVE

To evaluate how Brazilian orthopedists approach TKA, by means of a descriptive cross-sectional survey among orthopedists participating in the 39^th^ Brazilian Congress of Orthopedics and Traumatology (BCOT), in São Paulo, Brazil, and to verify how many were members of the Brazilian Society of Orthopedics and Traumatology (Sociedade Brasileira de Ortopedia e Traumatologia, SBOT).

## METHODS

During the 39^th^ BCOT, which was held in São Paulo, Brazil, between November 14 and 17, 2007, we applied a questionnaire to participating orthopedists, covering the methods they used and their preferences when performing TKA.

The protocol for this study had previously been submitted for assessment by SBOT and the Brazilian Society of Knee Surgery (Sociedade Brasileira de Cirurgia do Joelho, SBCJ). After analysis and approval by SBOT and SBCJ, the protocol was then submitted to and approved by the Research Ethics Committee of Universidade Federal de São Paulo - Escola Paulista de Medicina (Unifesp-EPM) (Decision Report No. 1611/06).

The questionnaires were distributed at three strategic points within the congress venue, at which three secretaries (who had previously been trained for this) handed them out randomly to congress participants. Completion of the questionnaire was voluntary. Questionnaires were also distributed at the entrance doors to the events room on the day when the specialty topic was the knee, and their availability was announced by the person chairing each event.

The questionnaire consisted of eleven objective questions (Annex 1). Questionnaires filled out completely by physicians who were orthopedists were included in the statistical analysis (except if they were foreign visitors). Questionnaires filled out by medical residents, physicians in other specialties and other types of healthcare professionals were excluded, as were any questionnaires that had been partially or incorrectly filled out.

The sample calculation was designed to estimate the proportion of Brazilian orthopedists that were performing TKA, with a proportion of 0.5, to result in the largest sample size possible. We used a confidence interval of 95% and a margin of error of 5%, thus arriving at a minimum sample requirement of 384 questionnaires.

## RESULTS

The total number of questionnaires distributed was 1200, and 939 of them were returned. Of these, 81 were excluded in accordance with the methodological criteria: 55 were incomplete; 14 had been answered by medical residents, five by foreign physicians, four by physicians in other specialties and three by undergraduate medical students. In accordance with the inclusion criteria, we had a final sample of 858 questionnaires, corresponding to 15.9% of the total number of congress attendees (5384) and 10.8% of the population of Brazil orthopedists in 2007 (7898).

**Graph 1.**
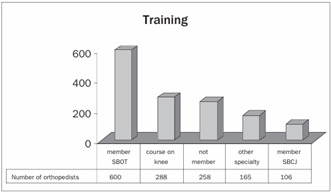
Training.

Around 70% of the questionnaires were answered by orthopedists who were titular members of SBOT, while 30% were not affiliated to SBOT. Out of the total of 858 participants, 33% had had training in knee surgery, 12% were members of SBCJ and 19% were working in other fields (Graph 1).

**Graph 2.**
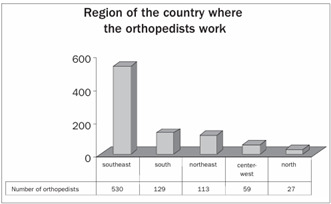
Number of orthopedists per region of Brazil.

With regard to the geographical region in which the respondents were working as orthopedists, we observed that 62% were in southeastern Brazil, while only 3% were in the north of the country (Graph 2).

**Graph 3.**
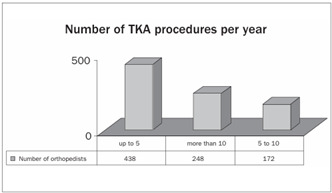
Number of total knee arthroplasty (TKA) procedures performed per year.

With regard to the number of TKA procedures performed per year, we observed that 51% of the participants carried out up to five and that only 29% carried out more than ten procedures per year (Graph 3).

**Graph 4.**
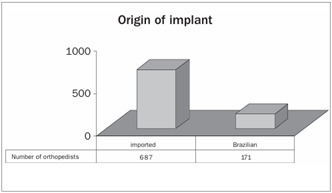
Type of implant used, with regard to manufacture.

The technique of full cementation of the implant was performed by 86% of the orthopedists, and only 3% chose to perform non-cemented implantation (Graph 4).

**Graph 5.**
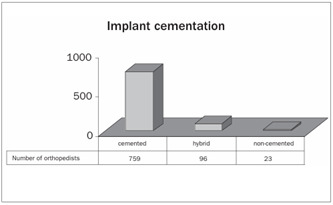
Type of cementation for implant.

**Graph 6.**
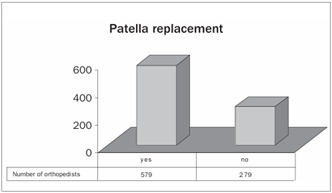
Replacement of the joint surface of the patella.

The joint surface of the patella was replaced by 67% of the orthopedists (Graph 5). The posterior cruciate ligament was preserved in 61% of the participants’ patients (Graph 6).

**Graph 7.**
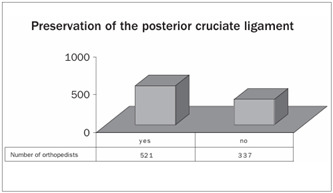
Type of implant, with regard to preservation of the posterior cruciate ligament.

Around 51% of the participants did not have any experience with the procedure of simultaneous bilateral TKA and only 9% of them thought that it was a good option for patients with advanced bilateral arthrosis of the knees (Graph 7).

**Graph 8.**
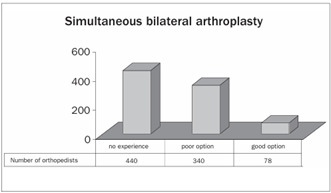
Simultaneous bilateral total knee arthroplasty.

Prophylactic antibiotics were administered by 60% of the participants for 48 hours, while 1% did not make use of antibiotic prophylaxis (Graph 8).

**Graph 9.**
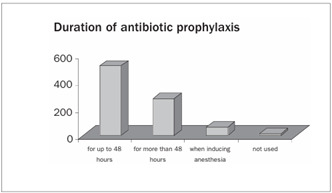
Use of antibiotic prophylaxis.

**Graph 10.**
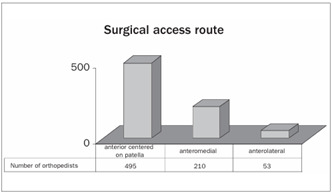
Preference regarding surgical access route.

The anterior access route centered on the patella was preferred by 65% of the interviewees. Only 7% preferred the anterolateral route (Graph 9). Postoperative suction drains were used by 93% of the orthopedists (Graph 10).

**Graph 11.**
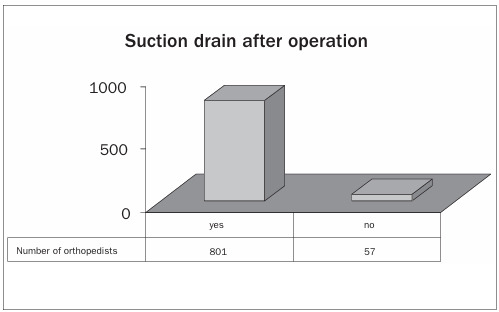
Use of suction drain after the operation.

**Graph 12.**
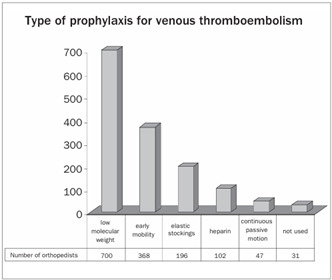
Use of prophylaxis for venous thromboembolism.

**Graph 13.**
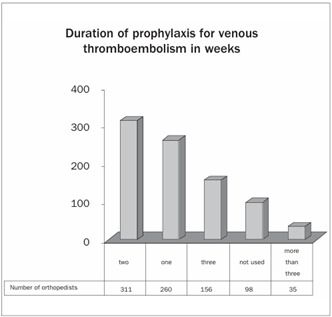
Duration of prophylaxis for venous thromboembolism in weeks.

With regard to prophylaxis for venous thromboembolism, 49% of the orthopedists made use of low molecular weight heparin. Only around 1.3% of the orthopedists said that they did not use any type of prophylaxis (Graph 11). Regarding the duration of prophylaxis for venous thromboembolism after discharge from hospital, 37% of the orthopedists used it for two weeks and 4% used it for more than three weeks (Graphs 12 and 13).

**Graph 14.**
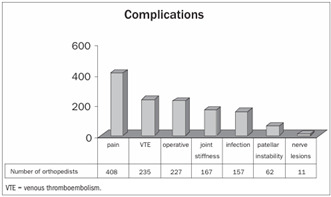
Main complications observed after the operation.

Among the more frequently observed complications, 32% reported persistent pain, 19% venous thromboembolism, 18% problems with the operative wound, 13% joint stiffness, 12% infection, 5% patellar instability and 1% neurological lesions (Graph 14).

## DISCUSSION

With the aim of making this study consistent with regard to the main aspects of approaches towards TKA, we drew up this questionnaire with consent from SBOT and SBCJ. The sample calculation was done while drawing up the study design, which was then submitted for evaluation and approval by the Research Ethics Committee of Escola Paulista de Medicina.

There were 5,384 orthopedists taking part in the congress, according to official data from SBOT.^13^ According to the sample calculation, we needed 358 questionnaires.

We distributed 1200 questionnaires by means of directly approaching congress participants who were passing by points that had previously been established, and during the lectures on the day for the specialty of the knee. The number of questionnaires that we distributed was large because we predicted the possibility of sample losses. We found that directly approaching the participants was the most effective means of sample collection, whereas the distribution during the lectures had an extremely low yield (0.3%), since those congress participants did not return the questionnaires. In the end, we obtained 858 valid completed questionnaires that were in accordance with the inclusion criteria. This number corresponded to 15.9% of the total number of orthopedists registered at the congress. This number was greater than the minimum sample size, which emphasizes that this study was representative in relation to Brazilian orthopedists’ opinions.

The total number of orthopedists included in this study took into consideration all the orthopedists who answered the questionnaire in accordance with the preestablished criteria, independent of whether they were members of SBOT. Around 70% of the participants in the study were members of SBOT. Only 12% were members of SBCJ. We do not have exact information on the number of professionals working in Brazil as orthopedists who are not members of SBOT.

Among the orthopedists who were not members of SBOT, around 58.5% were working in the southeastern region. This reinforces the need for better knowledge about these orthopedists who are not members of SBOT, in order to offer training and scientific refresher programs. Table 1 presents an overview of the number of orthopedists in Brazil and the number of orthopedists involved in this study, according to geographical regions of the country. Comparing data from SBOT and from this study, it can be seen that the results are representative of the geographical distribution of orthopedists in Brazil.

**Table 1.** Number of orthopedists in the Brazilian Society of Orthopedics and Traumatology (Sociedade Brasileira de Ortopedia e Traumatologia, SBOT) and at the congress, per region of Brazil

Region	Orthopedists in Brazil	Sample population at the congress (SBOT + others)
Southeast	4,184 (57%)	530 (62%)
South	1,346 (19%)	129 (15%)
Northeast	936 (13%)	113 (13%)
Center-West	569 (8%)	59 (7%)
North	206 (3%)	27 (3%)
Total	7,898 (100%)	858 (100%)

Despite the high cost, 80% of these Brazilian orthopedists preferred to use imported implants. Internationally, studies in the literature show a success rate of 90 to 95% from TKA procedures, considering a follow-up period of 10 to 15 years.^2^^,^^5^ There is a scarcity of data from Brazil, and it relates to the survival of imported implants.^6^^,^^7^ There is a growing need for studies comparing the survival of imported implants with the survival of Brazilian implants, considering that if the national implants were demonstrated to be viable, this would give rise to a significant decrease in costs.

There is a wealth of articles in the literature investigating what type of fixation would be ideal for TKA.^8^^,^^9^^,^^10^ There are authors who advocate the use of non-cemented TKA, and they cite the potential advantages of shorter duration of operations, greater technical ease in the event of surgical revision and longer survival for young patients.^8^ Nevertheless, there is no consensus regarding the best type of fixation for implants, and studies presenting better scientific evidence are needed. Nakama et al.^14^ have been developing a systematic review on this topic. In our study, we observed that 86% of the interviewees chose to use full cementation of the implant.

The published studies on replacement of the joint surface of the patella present controversies. The combination of large loads, multiple muscle actions, small contact surfaces and poor vascularization make the patella one of the greatest sources of complications following TKA.^15^ No Brazilian studies have shown any significant differences with regard to use or nonuse of the patellar component.^16^^,^^17^ Nizard et al.^18^ emphasized that it is difficult to reach a definitive conclusion because of the diversity of variables, such as the design of the component, experience of the surgeon and the degree and etiology of the patellar arthrosis. Among our respondents, 67% had performed procedures to replace the joint surface of the patella.

We found that the posterior cruciate ligament had been preserved in the procedures performed by 61% of our respondents. A review of the literature showed studies with excellent clinical-functional results from techniques for both preserving and replacing the posterior cruciate ligament.^19^^,^^20^ Some authors have stated that its preservation is important because of its neurosensory and biomechanical qualities, which improve gait and the proprioception of the knee.^6^ In a recent systematic review, Jacobs et al.^21^ did not find any conclusive results.

Approximately one third of the patients who undergo TKA suffer similar symptoms in the contralateral knee and thus require a bilateral approach.^22^ The literature shows that simultaneous bilateral TKA is a safe approach with a good cost-benefit relationship and a high degree of satisfaction among the patients.^22^^,^^23^^,^^24^ Despite these results, there is a constant focus on morbidity-mortality, which contributes towards viewing the procedure with reservations.^25^^,^^26^^,^^27^ In our study, we observed that 49% of the Brazilian orthopedists had not had any experience with this procedure, which confirms that this approach has not been disseminated much with the national sphere.

The use of antibiotic prophylaxis has been decreased to not more than 48 hours. Nelson et al.^28^ analyzed 358 patients who had received prophylaxis consisting of nafcillin or cefazoline for 48 hours. There was no significant difference in the prevalence of surgical infection after six weeks or a year of follow-up. Mauerhan et al.^29^ found that the patients who received antibiotics for periods longer than 24 hours presented increased risk of infection. In our study, we observed that 60% of the Brazilian orthopedists used antibiotics for a 48-hour period.

The medial parapatellar surgical access route is the one most reported in published studies in the Brazilian literature.^6^^,^^30^ Its potential disadvantages include destabilization of the extensor mechanism of the knee caused by changes in the vascular and neurological flow in the patella.^31^ With the aims of reducing the incidence of this complication and the need for surgical revision, new approaches have been proposed. There are few Brazilian studies on access routes for TKA.^32^ In our study, we observed a preference for the anterior access route centered on the patella, accounting for 65% of the cases.

There was a consensus regarding the use of suction drains among the Brazilian orthopedists, and they were used by 93% of them. Authors favoring the use of suction drains have stated that they reduce bleeding and hematoma formation.^33^^,^^34^ Those against their use have reported that drains increase blood losses and lead to a requirement for greater transfusion rates, as well as increasing the infection rates.^35^ Parker et al.^36^ and Esler et al.,^37^ concluded that further randomized studies are needed in order to refute or back the routine use of closed suction drains in orthopedic surgery.

Patients undergoing TKA are at a high risk of developing venous thromboembolism.^38^ The incidence of venous thromboembolism following TKA in which no prophylaxis was used ranges from 40% to 84%, and proximal thrombi are observed in 8% to 24% of these patients.^39^^,^^40^ Morrey et al.^27^ reported that the incidence of deep vein thrombosis and pulmonary thromboembolism was 1%. In a study on 1390 TKA procedures without using routine anticoagulant, Ansari et al.^41^ found that the incidence of fatal pulmonary thromboembolism was 0.7%. Venous thromboembolism was reported by 19% of the participants.

Pain in the knee following TKA is difficult to characterize, since it is generally multifactorial. It may compromise the results from the surgery and result in a functional level that is inferior to what there was before the operation. In patients in whom the patella is not replaced, one possible mechanism is contact between the degenerated patella and the femoral component. Another hypothesis is increased intraosseous pressure in situations of flexion greater than 15º, thereby impairing the circulation of subchondral bone. These two hypotheses suggest that replacement of the patella would eliminate the painful condition, although anterior pain in the knee has been found in 5% of the patients in whom the patella was replaced. Inappropriate alignment of the femoral and tibial components was seen in 10 to 30% of a series of radiological studies, and this may be a cause of persistent pain.^42^ Persistent pain following TKA procedures was cited by 32% of the orthopedists in our study.

Infection following TKA is a serious complication that puts the joint at risk and is occasionally life-threatening. The incidence of deep infection ranges from 1% to 5%. Superficial infection occurs in 10% to 20% of the cases.^7^^,^^43^^,^^44^ Around 12% of the participants in the present study reported that infection was the main problem during patient follow-up.

Problems with the operative wound were cited by 18% of the orthopedists. This shows the importance of the surgical access route and the manner in which soft tissue is manipulated. The incidence of problems with the operative wound ranges from 4.2% to 5%.^7^^,^^27^

Historically, the incidence of patellofemoral instability following TKA has ranged from 5% to 30%.^45^^,^^46^ Despite the advances in surgical techniques and implant design, complications involving the extensor mechanism and patellofemoral joint are the main causes of pain in the knee following TKA, as well as the greatest cause of the need for revision surgery in TKA cases.^47^^,^^48^ Cases involving patellofemoral instability were reported by 5% of the orthopedists in the present study.

Severe stiffness or arthrofibrosis following TKA is an unusual occurrence. Carvalho Júnior et al.^7^ described a stiffness rate of 7.5%. In our study, cases of joint stiffness were cited by 13% of the participants.

The real incidence of neurological lesions following arthroplasty is unknown. Among 10,361 arthroplasty procedures, Idusuyi et al.^49^ found 32 cases (0.3%) of lesions of the fibular nerve. Mont et al.^50^ found that the cumulative prevalence of peroneal nerve palsy after total knee arthroplasty was 0.58 per cent (74 out of 12,784 procedures), as reported in the literature. In a study in Brazil, Carvalho Júnior et al.^7^ did not describe any presence of neurological lesions. Only 1% of the orthopedists reported cases of neurological lesions.

## CONCLUSION

Based on the data obtained, we found that the majority of Brazilian orthopedists were members of SBOT and worked in the southeastern region of the country.

The Brazilian orthopedists presented concordant conduct regarding approaches towards TKA, in the following respects: use of imported cemented implants through an anterior access route centered on the patella, with replacement of the joint surface of the patella and preservation of the posterior cruciate ligament; bilateral TKA was performed as two operations at different times; antibiotic prophylaxis was used; and suction drains were used for 48 hours.

There was no consensus regarding prophylaxis for venous thromboembolism or the frequency of the main complications of TKA.

Implications for clinical practice and future studies

Based on the data from our study, we suggest that new comparative studies with good methodological quality should be carried out with the aim of obtaining conclusive evidence with regard to the following aspects of TKA:

1. Survival of Brazilian implants;
2. Cementation technique for implants;
3. Replacement of the joint surface of the patella;
4. Preservation of the posterior cruciate ligament;
5. Bilateral TKA: performed as a single operation or as two operations at different times;
6. Use of a closed suction drain;
7. Optimization of prophylaxis for thromboembolic events.

## References

[1] Bozic KJ, Kinder J, Meneghini RM, Zurakowski D, Rosenberg AG, Galante JO (2005). Implant survivorship and complication rates after total knee arthroplasty with a third-generation cemented system: 5 to 8 years followup. Clin Orthop Relat Res.

[2] Rand JA, Ilstrup DM (1991). Survivorship analysis of total knee arthroplasty. Cumulative rates of survival of 9200 total knee arthroplasties. J Bone Joint Surg Am.

[3] Ranawat CS, Flynn WF, Saddler S, Hansraj KK, Maynard MJ (1993). Long-term results of the total condylar knee arthroplasty. A 15-year survivorship study. Clin Orthop Relat Res.

[4] Ritter MA, Herbst SA, Keating EM, Faris PM, Meding JB (1994). Long-term survival analysis of a posterior cruciate-retaining total condylar total knee arthroplasty. Clin Orthop Relat Res.

[5] Font-Rodriguez DE, Scuderi GR, Insall JN (1997). Survivorship of cemented total knee arthroplasty. Clin Orthop Relat Res.

[6] Fuchs R, Mattuella F, Rabello LT (2000). Artroplastia total de joelho: avaliação a médio prazo: dois a dez anos [Total knee arthroplasty: midterm follow-up: two to ten years]. Rev Bras Ortop.

[7] Carvalho LH, Castro CAC, Gonçalves MBJ, Rodrigues LCM, Lopes FL, Cunha FVP (2006). Complicações de curto prazo da artroplastia total do joelho: avaliação de 120 casos [Short-term complications of knee total arthroplasty: evaluation of 120 cases]. Rev Bras Ortop.

[8] Whiteside LA (1994). Cementless total knee replacement. Nine- to 11-year results and 10-year survivorship analysis. Clin Orthop Relat Res.

[9] Rorabeck CH (1999). Total knee replacement: should it be cemented or hybrid?. Can J Surg.

[10] Luzo MVM, Navarro RD, Queiroz AAB, Carneiro M, Souza GA (1998). Artroplastia total do joelho. “Whiteside Ortholoc II” sem cimento. Avaliação clínica e radiográfica após dois anos de seguimento [Ortholoc II whiteside cementless total knee arthroplasthy. Clinical and radiologic evaluation after two years of follow-up]. Rev Bras Ortop.

[11] Instituto Brasileiro de Geografia e Estatística Indicadores sociodemográficos. Prospectivos para o Brasil 1991/2030.

[12] Pires RES, Fernandes HJA, Belloti JC, Balbachevsky D, Faloppa F, Reis FB (2006). Como são tratadas as fraturas diafisárias fechadas do fêmur no Brasil? Estudo transversal [How are closed femoral diaphyseal fractures treated in Brazil? A cross-sectional study]. Acta Ortop Bras.

[13] Sociedade Brasileira de Ortopedia e Traumatologia (2007). Entre os melhores do mundo. Jornal da SBOT.

[14] Nakama GY, Peccin MS, Almeida GJM, Lira OA, Navarro RD (2006). Cemented vs cementless in total knee arthroplasty for osteoarthrosis and other non-traumatic diseases. (Protocol). Cochrane Database of Systematic Reviews.

[15] Ayers DC, Dennis DA, Johanson NA, Pellegrini VD (1997). Instructional Course Lectures, The American Academy of Orthopaedic Surgeons - Common complications of total knee arthroplasty. J Bone Joint Surg Am.

[16] Turqueto L, Villardi A, Leite ER, Palma IM, Tejada JVH (1994). Artroplastia total do joelho com e sem substituição da patela [Total knee arthroplasty with and without resurfacing of the patella]. Rev Bras Ortop.

[17] Barbosa REA, Pacheco LRL, Alencar PGC (2003). Influência do grau de artrose femoropatelar no resultado da artroplastia total do joelho, sem o uso do componente patelar [Influence of the femoropatellar osteoarthritis degree on the outcome of total knee arthroplasty without patellar resurfacing]. Rev Bras Ortop.

[18] Nizard RS, Biau D, Porcher R (2005). A meta-analysis of patellar replacement in total knee arthroplasty. Clin Orthop Relat Res.

[19] Scott RD, Volatile TB (1986). Twelve years’ experience with posterior cruciate-retaining total knee arthroplasty. Clin Orthop Relat Res.

[20] Villardi AM, Veiga LT, Franco JS (2005). Análise da marcha pós-artroplastia total do joelho com e sem preservação do ligamento cruzado posterior [Gait analysis after total knee arthroplasty with and without posterior cruciate ligament preservation]. Rev Bras Ortop.

[21] Jacobs WC, Clement DJ, Wymenga AB (2005). Retention versus sacrifice of the posterior cruciate ligament in total knee replacement for treatment of osteoarthritis and rheumatoid arthritis. Cochrane Database Syst Rev.

[22] Ivory JP, Simpson AH, Toogood GJ, McLardy-Smith PD, Goodfellow JW (1993). Bilateral knee replacements: simultaneous or staged?. J R Coll Surg Edinb.

[23] Walmsley P, Murray A, Brenkel IJ (2006). The practice of bilateral, simultaneous total knee replacement in Scotland over the last decade. Data from the Scottish Arthroplasty Project. Knee.

[24] Reuben JD, Meyers SJ, Cox DD, Elliott M, Watson M, Shim SD (1998). Cost comparison between bilateral simultaneous, staged, and unilateral total joint arthroplasty. J Arthroplasty.

[25] Brotherton SL, Roberson JR, de Andrade JR, Fleming LL (1986). Staged versus simultaneous bilateral total knee replacement. J Arthroplasty.

[26] Buscemi MJ, 2nd Page BJ, Swienckowski J (1989). Unilateral versus bilateral simultaneous arthroplasties of the lower extremities. J Am Osteopath Assoc.

[27] Morrey BF, Adam RA, Ilstrup DM, Bryan RS (1987). Complications and mortality associated with bilateral or unilateral total knee arthroplasty. J Bone Joint Surg Am.

[28] Nelson CL, Green TG, Porter RA, Warren RD (1983). One day versus seven days of preventive antibiotic therapy in orthopedic surgery. Clin Orthop Relat Res.

[29] Mauerhan DR, Nelson CL, Smith DL (1994). Prophylaxis against infection in total joint arthroplasty. One day of cefuroxime compared with three days of cefazolin. J Bone Joint Surg Am.

[30] Mestriner LA, Laredo J (1993). Artroplastia total do joelho em osteoartrose [Total knee replacement in osteoarthritis]. Rev Bras Ortop.

[31] Fauré BT, Benjamin JB, Lindsey B, Volz RG, Schutte D (1993). Comparison of the subvastus and paramedian surgical approaches in bilateral knee arthroplasty. J Arthroplasty.

[32] Bem AAM, Gomes JLE, Marczyk LRS (2003). Abordagens cirúrgicas para artroplastia primária total de joelho [Surgical approaches for primary total knee arthroplasty]. Rev Bras Ortop.

[33] Holt BT, Parks NL, Engh GA, Lawrence JM (1997). Comparison of closed-suction drainage and no drainage after primary total knee arthroplasty. Orthopedics.

[34] Willemen D, Paul J, White SH, Crook DW (1991). Closed suction drainage following knee arthroplasty: Effectiveness and risks. Clin Orthop Relat Res.

[35] Minnema B, Vearncombe M, Augustin A, Gollish J, Simor AE (2004). Risk factors for surgical-site infection following primary total knee arthroplasty. Infect Control Hosp Epidemiol.

[36] Parker MJ, Livingstone V, Clifton R, McKee A (2007). Closed suction surgical wound drainage after orthopaedic surgery. Cochrane Database Syst Rev.

[37] Esler CN, Blakeway C, Fiddian NJ (2003). The use of a closed-suction drain in total knee arthroplasty. A prospective, randomised study. J Bone Joint Surg Br.

[38] Westrich GH, Haas SB, Mosca P, Peterson M (2000). Meta-analysis of thromboembolic prophylaxis after total knee arthroplasty. J Bone Joint Surg Br.

[39] Grady-Benson JC, Oishi CS, Hanson PB, Colwell CW, Otis SM, Walker RH (1994). Postoperative surveillance for deep venous thrombosis with duplex ultrasonography after total knee arthroplasty. J Bone Joint Surg Am.

[40] Garino JP, Lotke PA, Kitziger KJ, Steinberg ME (1996). Deep venous thrombosis after total joint arthroplasty. The role of compression ultrasonography and the importance of the experience of the technician. J Bone Joint Surg Am.

[41] Ansari S, Warwick D, Ackroyd CE, Newman JH (1997). Incidence of fatal pulmonary embolism after 1,390 knee arthroplasties without routine prophylactic anticoagulation, except in high-risk cases. J Arthroplasty.

[42] Muoneke HE, Khan AM, Giannikas KA, Hägglund E, Dunningham TH (2003). Secondary resurfacing of the patella for persistent anterior knee pain after primary knee arthroplasty. J Bone Joint Surg Br.

[43] Tsukayama DT, Gustilo R, Fu FH, Harner CD, Vince KG (1994). Infected total knee arthroplasty. Knee surgery.

[44] Kim S, Losina E, Solomon DH, Wright J, Katz JN (2003). Effectiveness of clinical pathways for total knee and total hip arthroplasty: literature review. J Arthroplasty.

[45] Merkow RL, Soudry M, Insall JN (1985). Patellar dislocation following total knee replacement. J Bone Joint Surg Am.

[46] Mochizuki RM, Schurman DJ (1979). Patellar complications following total knee arthroplasty. J Bone Joint Surg Am.

[47] Malo M, Vince KG (2003). The unstable patella after total knee arthroplasty: etiology, prevention, and management. J Am Acad Orthop Surg.

[48] Lewonowski K, Dorr LD, McPherson EJ, Huber G, Wan Z (1997). Medialization of the patella in total knee arthroplasty. J Arthroplasty.

[49] Idusuyi OB, Morrey BF (1996). Peroneal nerve palsy after total knee arthroplasty. Assessment of predisposing and prognostic factors. J Bone Joint Surg Am.

[50] Mont MA, Dellon AL, Chen F, Hungerford MW, Krackow KA, Hungerford DS (1996). The operative treatment of peroneal nerve palsy. J Bone Joint Surg Am.

